# Steady-state solutions of split beams in electron storage rings

**DOI:** 10.1038/s41598-022-22857-y

**Published:** 2022-11-01

**Authors:** Marc Andre Jebramcik, Shaukat Khan, Wolfram Helml

**Affiliations:** Department of Physics, Center for Synchrotron Radiation (DELTA), TU Dortmund University, 44227 Dortmund, Germany

**Keywords:** Physics, Statistical physics, thermodynamics and nonlinear dynamics

## Abstract

Recently, a novel operation method for synchrotron light sources with transversely split beams has been explored to fulfill the rising demand for flexible and high-throughput X-ray sources required in such diverse fields as time-resolved X-ray spectroscopy, molecular chemistry in organic cells, high-resolution medical imaging, quantum materials science or sustainable energy research. Within that novel operation mode, additional stable regions are produced in the horizontal phase space by operating an electron storage ring on a resonance that is driven by the nonlinear sextupole or octupole magnets. In the longitudinal phase space, a similar split can be produced by introducing an oscillation of the synchrotron phase via a modulation of the phase of the radiofrequency resonator. Strong radiation damping in electron storage rings, however, has to be overcome before additional regions in phase space can become populated by particles and form stable islands. This damping mechanism changes the dynamics of the system and causes diffusion between the different islands in phase space, raising the question what kind of equilibrium state exists in the asymptotic temporal limit. In this paper, a finite-differences approximation in rotating action-angle coordinates is used to solve the Vlasov–Fokker–Planck equation and to study the obtained equilibrium states for the longitudinal as well as the transverse case. The number of solution vectors and the magnitude of the corresponding singular values of the matrix of the underlying finite-differences equation are used as abstract indicators to define the required parameter set that provides stable additional beamlets. As a consequence, the beamlets have a stability that is close to that of the main beam in terms of diffusion caused by the radiation damping and quantum excitation.

## Introduction

**Figure 1:**
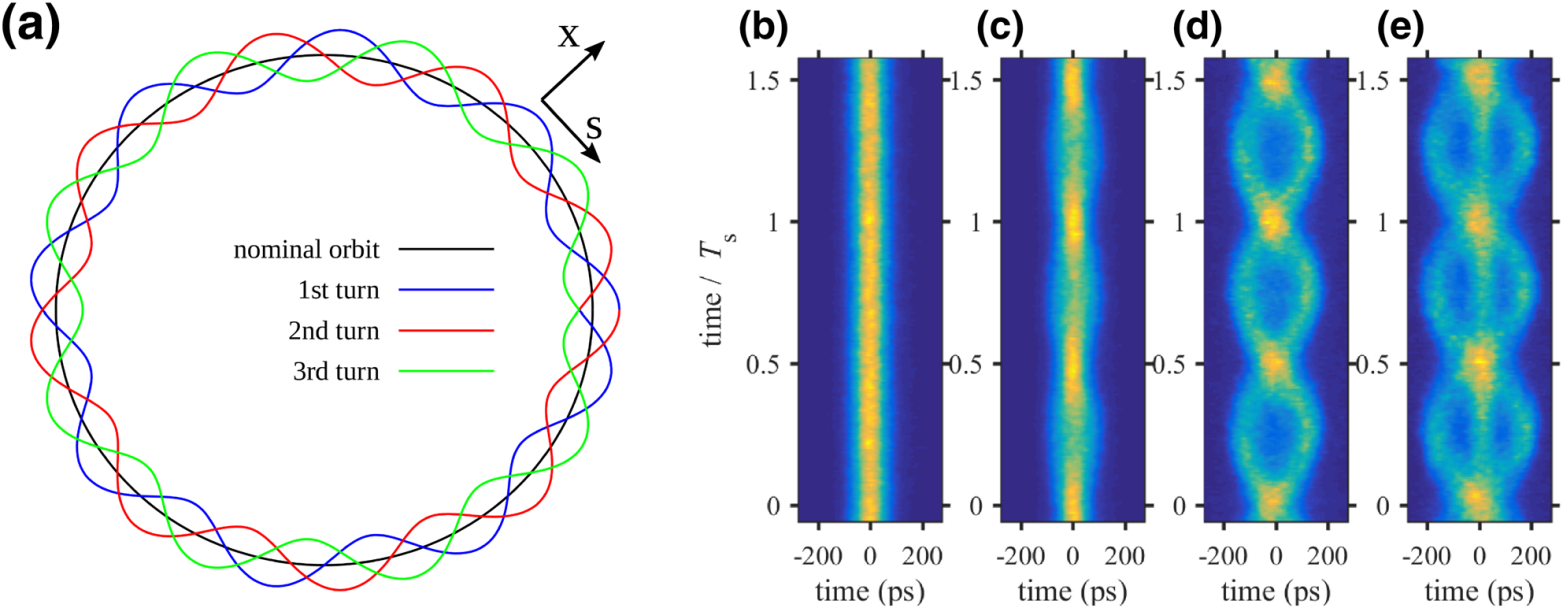
**(a)** Schematic top view of the orbit in a storage ring when operated on a horizontal third-order resonance. While the nominal orbit (black) closes after one revolution, the three additional orbits of the resonance islands merge into each other leading to a fully closed orbit after three revolutions in the machine. The orbit excursion in the horizontal coordinate *x* is strongly exaggerated. **(b–e)** Dual-scan streak camera images for different RF phase modulation parameters close to the second integer resonance ($\omega _\mathrm {m} \approx 2 \omega _\mathrm {s}$) at DELTA. The color-coded radiation intensity, which corresponds to the electron density in the bunch, is shown as function of time on a picosecond (abscissa) and a microsecond scale (ordinate, here in units of the synchrotron period $T_\mathrm {s}$). While keeping the modulation frequency constant, the synchrotron frequency was tuned by changing the RF voltage. From left to right: **(b)** modulation off, **(c)** $\omega _\mathrm {m} > 2 \omega _\mathrm {s}$, **(d)** two-island regime with $\omega _\mathrm {m}$ close to $2 \omega _\mathrm {s}$, **(e)** three-island regime with $\omega _\mathrm {m} < 2 \omega _\mathrm {s}$.

Besides the development of free-electron lasers as state-of-the-art X-ray sources, also synchrotron light sources have seen a tremendous boost in performance in recent years. Today, fourth-generation electron storage rings, like MAX IV^1^ in Lund, Sweden, or the new ESRF-EBS^2^ in Grenoble, France, provide unprecedentedly low beam emittance and thus offer the potential for ultrabright and coherent synchrotron radiation. These highly specialized machines are based on different versions of multi-bend achromatic lattice designs and combine MHz repetition rates for simultaneous experiments at multiple beamlines within a wavelength range from the THz to the hard X-ray regime. They can serve as broadband sources for ultrashort pulse generation and as monoenergetic devices for high-energy-resolution X-ray diffraction, imaging or spectroscopy, or for a combination of these schemes. Improved electron beam characteristics lead to a growing demand for experiments by research groups from such different areas of science as structural biology, material science and photochemistry. New methods for data analysis on-the-fly with techniques from the machine learning community open the chance of utilizing novel and previously unconsidered operation modes of synchrotron light sources that enhance the flexibility of the facilities while allowing multiple measurements at the same time. One candidate for such a modern operation mode is to set the betatron tune close to a resonance of the storage ring, introducing additional electron orbits, so-called transverse resonance island buckets (TRIBs) that have recently been applied in electron storage rings to achieve highly desirable goals like the production of helicity-flipped synchrotron light^3^, a hybrid filling mode to provide high-intensity single bunches on one orbit for timing users while providing a dense, high-brightness bunch train on another orbit^4^. Operation with transversely split beams has been used in the CERN SPS to provide slow extraction for more than a decade^5^. A schematic top view of the orbit with three additional transverse resonance islands is given in Fig. 1a.

Nevertheless, the novel operation mode is still actively investigated and not fully understood in terms of the feasibility since it requires the careful operation of the storage ring close to a nonlinear resonance to produce the additional stable areas in the transverse phase space to accommodate supplementary beamlets. The operation close to these nonlinear resonances was long considered destructive for the beam, and the usual procedure is to place the machine’s working point cautiously between resonances. One of the uncertainties of the operation mode with resonance islands is the potential diffusion of particles between the islands in electron machines, since emission of synchrotron radiation in combination with the resulting quantum excitation may cause particle exchange between the islands. Such a behaviour can be observed at BESSY II, Berlin, Germany, when it is operated close to a third-order resonance driven by sextupoles. The diffusion process is important to understand and cautiously optimize the injection procedure into the additional orbits, where poorly placed islands in fixed points that continuously lose particles to, e.g., the centre orbit would make matters worse.

Because the description of the nonlinear dynamics in the transverse phase space seems complicated considering the large number of nonlinear magnets that may contribute to the particle dynamics, this paper will derive the required principles for the longitudinal phase space in the presence of radiofrequency (RF) phase modulation first and then apply the same principles to the transverse phase space. These investigations are fully based on solving the Vlasov-Fokker-Planck (VFP) equation on a grid. This approach is superior to tracking method or to solving the time-dependent VFP equation since these approaches require a long simulation time to reach equilibrium and it is unclear whether multiple equilibrium states may exist. Deriving a time-independent Hamiltonian instead allows all possible orthogonal equilibrium states to be computed directly.

## RF phase modulation

An RF phase modulation is commonly used at light sources like DELTA at TU Dortmund University^6,7^ to enhance the beam lifetime by increasing the phase space volume and thus reducing the loss rate due to the Touschek effect. In addition, longitudinal coupled-bunch instabilities excited by, e.g., the RF cavity are effectively suppressed. The phase modulation modifies the RF wave according to

1 $$\begin{aligned} U_\text {mod} = U_0 \sin (\omega _\text {RF}t + A_\text {m}\sin \omega _\text {m}t) \end{aligned}$$

with the peak RF voltage $U_0$, the RF angular frequency $\omega _\text {RF}$, the modulation amplitude $A_\text {m}$, and the modulation frequency $\omega _\text {m}$. If the modulation frequency is chosen close to an integer multiple of the synchrotron frequency $\omega _\text {s}$, i.e., $\omega _\text {m} \approx n \omega _\text {s}$ with $n \in {\mathbb {N}}^+$, resonant excitation is achieved and islands, additional stable fixed points besides the bunch centre, are obtained with a cautiously selected modulation frequency and amplitude as is displayed by the streak-camera pictures given in Fig. 1b-e.

### Hamiltonian at an arbitrary integer resonance

Neglecting the localized nature of the RF cavity, the Hamiltonian for the longitudinal phase space with a single RF cavity and active RF phase modulation reads^8–10^

2 $$\begin{aligned} H (\phi , \delta , t) = \frac{1}{2} \omega _\text {s} \delta ^2 + \frac{- \omega _\text {s}}{\cos \phi _\text {s}} \left( \phi \sin \phi _\text {s} \right. \left. +\cos (\phi _\text {s}+\phi +A_\text {m}\sin (\omega _\text {m}t) ) \right) \, \end{aligned}$$

with the normalized momentum coordinate $\delta =({\Delta E}/{E_0}) {h \eta }/{Q_\text {s}}$ and the phase angle $\phi$ being measured with respect to the synchronous phase angle $\phi _\text {s}$. Here, *h* is the harmonic number, $\eta$ is the slippage factor, $Q_\text {s}$ is the synchrotron tune, and $\Delta E$ is the energy deviation from the nominal energy $E_0$. The Hamiltonian is quite unhandy and time-dependent making it difficult to identify potential fixed points in phase space. In a first step, a transformation into action-angle coordinates $(\Phi ,I)$ is performed via the generating function $F_1(\phi ,\Phi )=-(\phi ^2/2)\tan \Phi$ leading to the substitutions $\delta = -\sqrt{2I}\sin \Phi$, $\phi =\sqrt{2I}\cos \Phi$. Since this still does not eliminate the time dependence of the Hamiltonian, a second transformation into a rotating coordinate system $(\Psi , J)$ is performed via the generating function^8,9,11^ $F_2(\Phi ,J )=J (\Phi - \omega _\text {m}t/n - \pi /(2n))$ of second kind to achieve the substitutions $I=J$ and $\Phi = \Psi +\omega _\text {m}t/n+\pi /(2n)$. After time averaging to eliminate time-varying terms and a small-angle approximation with regard to the perturbation term, one reaches the final time-independent Hamiltonian^8,11^

3 $$\begin{aligned} K(\Psi ,J) = \left( \omega _\text {s}-\frac{\omega _\text {m}}{ n} \right) J - \frac{\omega _\text {s}}{16} J^2 + c(n) (2J)^{\frac{n}{2}} \cos {n\Psi }. \end{aligned}$$

with the scalar constant

4 $$\begin{aligned} c(n) = {\left\{ \begin{array}{ll}A_\text {m}\omega _\text {s} \frac{(-1)^{\frac{n+1}{2}}}{2^n n!} &{} {\text { if}} \, { n} \, {\text {is odd,}} \\ A_\text {m}\omega _\text {s} \tan \phi _\text {s} \frac{(-1)^{\frac{n+2}{2}}}{2^n n!} &{} {\text { if}} \, { n} \, {\text {is even.}} \end{array}\right. } \, \end{aligned}$$

Note that the absolute value of *c*(*n*) drops rapidly with increasing *n*.

### Stable fixed points in longitudinal phase space

The Hamiltonian *K* in Eq. (3) can easily be investigated for stable fixed points (SFPs) by solving

5 $$\begin{aligned} \left( \frac{\partial K}{\partial \Psi }, \frac{\partial K}{\partial J}\right) \bigg |_{J=J_\text {FP},\Psi =\Psi _\text {FP}}=(0,0) \end{aligned}$$

for $J_\text {FP}$ and $\Psi _\text {FP}$. The stability of the fixed points can be checked by inspecting the Hessian

6 $$\begin{aligned} {\mathbf {H}}= \begin{pmatrix} \frac{\partial ^2 K}{\partial \Psi ^2} &{} \frac{\partial ^2 K}{\partial \Psi \partial J} \\ \frac{\partial ^2 K}{\partial \Psi \partial J} &{} \frac{\partial ^2 K}{\partial J^2} \end{pmatrix} \bigg |_{J=J_\text {FP}, \Psi =\Psi _\text {FP}} \end{aligned}$$

to be positive definite, which implies eigenvalues on the complex unit circle and consequently Ljapunov stability. Unstable fixed points (UFPs) that are also obtained via Eq. (5) do not yield a positive definite Hessian.

### Fixed points for ${n=2}$ and ${n=3}$

**Figure 2:**
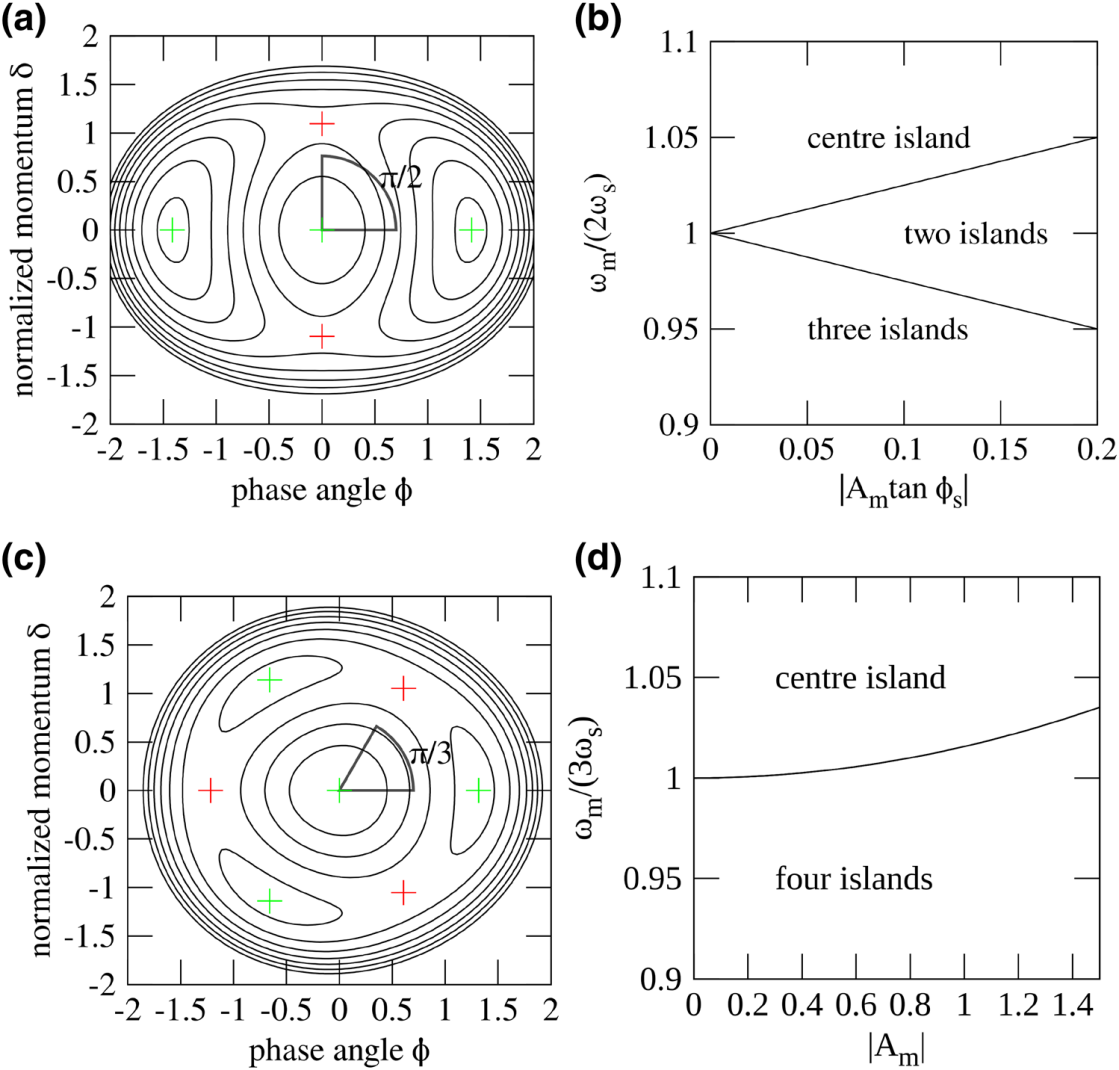
**(a)** Three-islands regime at the second integer resonance $n=2$ with $\omega _\text {m}/(2\omega _\text {s})=0.9$ and $A_\text {m}\tan \phi _\text {s}=0.1$. The green markers indicate the SFPs while the red markers indicates the UFPs. **(b)** The diagram shows for which combinations of the modulation amplitude and frequency at the second integer resonance ($n=2$) either only the centre island, two lateral islands, or three islands (centre island and two lateral islands) exist. **(c)** Four-islands regime at the third integer resonance $n=3$ with $\omega _\text {m}/(3 \omega _\text {s})=0.9$ and $A_\text {m}=0.1$. The green markers indicate the SFPs while the red markers indicate the UFPs. **(d)** The diagram displays for which combination of modulation amplitude and modulation frequency at the third integer resonance ($n=3$) only the centre island or four islands (centre island and three lateral islands) exist.

The results of the stability analysis of the fixed points for $n=2$ and $n=3$ are given in the following for illustrative purposes, since for these cases the Vlasov-Fokker-Planck equation is going to be solved later on. Figure 2a,c show examples in which the stable fixed points are marked in green while the unstable fixed points are indicated in red. The fixed points for various resonances are well known^8–10,12^. For $n=2$ for example, they are located at the actions^8,11^

7 $$\begin{aligned} J_\text {SFP}&= 8\left| 1-\frac{\omega _\text {m}}{2 \omega _\text {s}} \right| +2|A_\text {m}\tan \phi _\text {s}| , \end{aligned}$$

8 $$\begin{aligned} J_\text {UFP}&= 8\left| 1-\frac{\omega _\text {m}}{2 \omega _\text {s}} \right| -2|A_\text {m}\tan \phi _\text {s}| . \end{aligned}$$

These expressions indicate a bifurcation condition for the islands to exist, i.e., $4|1-\omega _\text {m}/(2 \omega _\text {s})| \ge |A_\text {m}\tan \phi _\text {s}|$. The SFPs and UFPs alternate in terms of the angle, and the angular distance is $\Delta \Psi = \pi /2$ as can also be seen in Fig. 2a. The centre island may also vanish depending on the RF phase modulation parameters, i.e., it becomes an UFP. This behaviour is shown in the bifurcation diagram in Fig. 2b. Calculations for $n=2$ have shown that the modulation amplitude $A_\text {m}$ has to exceed the value $A_\text {m}\ge 4 \gamma _\text {d} \sqrt{1+\omega _\text {m}^2/(16\omega _\text {s}^2 \gamma _\text {d}^2)}/(\omega _\text {s}|\tan \phi _\text {s}|)$ with the longitudinal damping rate $\gamma _\text {d}$ to overcome radiation damping in the bunch centre to clear out the centre UFP^8,13^; however, this threshold is not easily calculable for an arbitrary resonance *m*.

The third integer resonance behaves similarly as the second integer resonance. The stability between the lateral islands changes alternately in terms of angle as shown in Fig. 2c. The angular distance between the stable and unstable fixed points is $\Delta \Psi = \pi /3$. The action of the fixed points is^9,11,12^

9 $$\begin{aligned} J_\text {SFP}&= \left( \frac{A_\text {m}}{ \sqrt{8}} + \sqrt{\frac{A_\text {m}^2}{8}+8\left( 1- \frac{\omega _\text {m}}{3 \omega _\text {s}} \right) }\right) ^2 , \end{aligned}$$

10 $$\begin{aligned} J_\text {UFP}&= \left( \frac{A_\text {m}}{ \sqrt{8}} - \sqrt{\frac{A_\text {m}^2}{8}+8\left( 1- \frac{\omega _\text {m}}{3 \omega _\text {s}} \right) }\right) ^2 . \end{aligned}$$

The bifurcation diagram in Fig. 2d directly results from the expression within the square root in Eqs. (9)–(10), i.e., $\omega _\text {m}/(3 \omega _\text {s}) \le 1 + A_\text {m}^2/64$. The stability of the centre island, however, is always guaranteed since high-order amplitude detuning terms are not present, only the face area of the centre island may vary. It is worth mentioning that the $\Delta \Psi =\pi /3$ distance between stable and unstable fixed points does not apply at a small frequency window close to the resonance frequency $1 \le \omega _\text {m}/(3 \omega _\text {s}) \le 1 + A_\text {m}^2/64$. In this region, stable and unstable fixed points share the same $\Psi$ coordinate with the stable (unstable) fixed point having the larger (smaller) action.

## Vlasov–Fokker–Planck equation in the presence of RF phase modulation

The Vlasov–Fokker–Planck (VFP) equation is a partial differential equation describing the evolution of the density function $\rho$ in phase space under the influence of quantum excitation and radiation damping. The general VFP equation using the Hamiltonian *H* from Eq. (2) in the longitudinal phase space reads^9^

11 $$\begin{aligned} \frac{\partial \rho }{\partial t} + \left[ \rho ,H \right] _{\phi ,\delta } = \frac{\partial }{\partial \delta } \left( 2 \gamma _\text {d} \rho \delta +D \frac{\partial \rho }{\partial \delta } \right) \, \end{aligned}$$

with $\left[ \rho ,H\right] _{\phi ,\delta }$ being the Poisson brackets with respect to the coordinate $\phi$ and the conjugate momentum $\delta$, $\gamma _\text {d}$ being the longitudinal damping coefficient and *D* is the diffusion coefficient. With the same coordinate transformation as in the previous section, the VFP equation in rotating action-angle coordinates with *K* taken from Eq. 3 reads^9,11^

12 $$\begin{aligned} \left[ \rho ,K \right] _{\Psi ,J} =2 \gamma _\text {d} \left( \rho + J \frac{\partial \rho }{\partial J} \right) +D\left( \frac{\partial \rho }{\partial J} +J \frac{\partial ^2 \rho }{\partial J^2}+\frac{1}{4J}\frac{\partial ^2 \rho }{\partial \Psi ^2} \right) \, \end{aligned}$$

after time averaging and elimination of the oscillating terms. The term $\partial \rho /\partial t$ is also dropped since the goal is to obtain a steady-state solution in rotating action-angle coordinates. In the absence of RF phase modulation, the VFP equation is solved by $\rho (J)=k \exp \left( - 2 \gamma _\text {d} J/D \right)$ with $k \in {\mathbb {R}}^+$ and $D=2\gamma _\text {d} \sigma ^2$. This expression for $\rho$ simply corresponds to the natural distribution in $(\phi ,\delta )$ space. The bunch length $\sigma$ is the same in $\phi$ and $\delta$ due to the choice of coordinates. When the RF phase modulation is switched on, numerical calculations have to be performed to obtain $\rho$ as is shown in the following. Diffusion between the islands due to the Touschek effect or RF voltage jitters is not considered.

### The VFP equation on a grid

**Figure 3:**
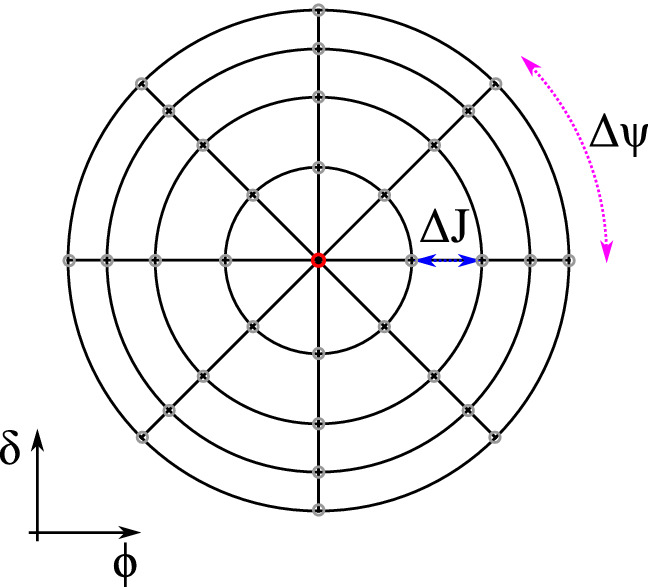
The $(\phi ,\delta )$ phase space is discretized in a time-dependent action-angle frame $(\Psi ,J)$ with $N_J=4$ and $N_\Psi =8$ knots in this particular example. The centre at $J=0$ (red marker) is not part of the grid to avoid singularities when solving the VFP equation. The first ring of equal action is at a distance $J=\Delta J/2$ from the centre of the grid.

To solve the VFP equation numerically, the phase space is discretized in the action-angle coordinates with $N_\Psi$ knots in angle and $N_J$ knots in action as sketched in Fig. 3. The increments between adjacent grid knots are fixed at $\Delta \Psi$ and $\Delta J$ in the two coordinates. Since the action-angle coordinates are a nonlinear transformation of the Cartesian $(\phi ,\delta )$ plane, the grid appears non-equidistant in $(\phi ,\delta )$ space. With the innermost ring at $J=\Delta J/2$, the singularity appearing in the VFP equation at the coordinate origin $J=0$ is avoided (singularity due to division by $J=0$). Furthermore, $\rho$ at the edge of the domain, in which the VFP equation is solved, is set to zero. This is not arbitrary, because in reality the energy acceptance will cause any density function to drop to zero at a sufficiently large action.

A feature of the discretization in action-angle coordinates is the determinant of the Jacobian being equal to unity, i.e., $\iint _G f(\delta ,\phi )\text {d}\delta \text {d}\phi =\iint _G f(\delta (\Psi ,J)),\phi (\Psi ,J))\text {d}\Psi \text {d}J$ for a function *f* on a given domain *G*. With a second-order approximation of the first partial derivative and a first-order approximation of the second-order partial derivative with respect to angle and action, the VFP equation modifies locally to^11^

13 $$\begin{aligned}\rho _{i,j}\left( 2 \gamma _\text {d} - \frac{2DJ_{i,j}}{(\Delta J)^2}- \frac{D}{2J_{i,j}(\Delta \Psi )^2} \right) + \rho _{i+1,j} \left( \frac{D}{4J_{i,j}(\Delta \Psi )^2} - \frac{1}{2\Delta \Psi } \frac{\partial K}{\partial J} \right) + \rho _{i-1,j} \left( \frac{D}{4J_{i,j}(\Delta \Psi )^2} + \frac{1}{2\Delta \Psi } \frac{\partial K}{\partial J} \right) \nonumber \\&+ \rho _{i,j+1} \left( \frac{DJ_{i,j}}{(\Delta J)^2} + \frac{1}{2\Delta J} \frac{\partial K}{\partial \Psi } + \frac{2\gamma _\text {d} J_{i,j}}{2\Delta J}+\frac{D}{2\Delta J} \right) + \rho _{i,j-1} \left( \frac{DJ_{i,j}}{(\Delta J)^2} - \frac{1}{2\Delta J} \frac{\partial K}{\partial \Psi } - \frac{2\gamma _\text {d} J_{i,j}}{2\Delta J}-\frac{D}{2\Delta J} \right) = 0 \end{aligned}$$

in the finite-differences approach. Here, $\rho _{i,j}$ is the value of the density function at the coordinates $\Psi =i \Delta \Psi$ and $J=(1/2+j)\Delta J$. The equation can be rearranged to obtain the homogeneous equation

14 $$\begin{aligned} {\mathbf {M}}{\mathbf {x}}={\mathbf {0}} \end{aligned}$$

with ${\mathbf {x}}$ being the solution vector of length $l=N_\Psi N_J$ containing the different $\rho _{ij}$ at the grid knots, and ${\mathbf {M}}$ is the highly sparse matrix of size $l \times l$.

For $n\ge 2$, the symmetry of the system can be exploited: Eq. (13) is symmetric if $\Psi$ is translated by the angle $2\pi /n$. Hence, with cyclic boundary conditions, the domain can be restricted to $2\pi /n$, which heavily reduces the number of required grid knots in the $\Psi$ coordinate.

### Solving the VFP equation

Since Eq. (14) is homogeneous, a vanishing determinant of the matrix ${\mathbf {M}}$ is required to have non-trivial solutions besides the trivial solution (${\mathbf {x}}=0$). In order to find the solutions to Eq. (14) (the kernel of ${\mathbf {M}}$), a singular value decomposition (SVD) is performed that reads

15 $$\begin{aligned} {\mathbf {M}} = {\mathbf {V}}{\Sigma } {\mathbf {U}}^\mathrm {T} \end{aligned}$$

with ${\Sigma }=\text {diag}(\sigma _1, ..., \sigma _l)$ being a diagonal matrix containing the singular values $\sigma _i$ in descending order and ${\mathbf {V}}$ and ${\mathbf {U}}$ being non-unique orthonormal matrices. At this point, it is assumed that $N_0$ singular values in ${\Sigma }$ are zero. The kernel of ${\mathbf {M}}$ corresponds to the span of the $N_0$ column vectors ${\mathbf {u}}_i$ with $i \in (1,...,N_0)$ of the matrix ${\mathbf {U}}$ associated with the $N_0$ singular values that are zero. The span of these orthonormal vectors constitutes the desired solutions for ${\mathbf {x}}$ in Eq. (14). Hence, the solutions are

16 $$\begin{aligned} {\mathbf {x}} = \sum _{i=1}^{N_0} b_i {\mathbf {u}}_i \end{aligned}$$

with the scalar coefficients $b_i$. Finding these solutions via iterative algorithms like Gauss-Seidel or Jacobi iteration would not lead to all solutions with the final result heavily depending on the initial solution vector. This is the main reason why these iterative methods are avoided in addition to their poor performance. Our approach also overcomes the downsides of particle tracking: With the inclusion of some random changes of the momentum to simulate quantum excitation, particle tracking takes too much computation time to reach equilibrium and the final state depends on the initial condition. In addition, only one solution vector is obtained even if multiple solutions may exist.

A drawback of solving the matrix equation directly via SVD is the potential existence of non-physical solutions for which the solution vector ${\mathbf {x}}$ contains positive as well as negative values. For a solution to be physical, all vector entries have to be either positive or negative, since a fully negative vector ${\mathbf {x}}$ can be multiplied by $-1$ to obtain positive values. The coefficients $b_i$ may therefore also be all negative. It is important to note that a base vector ${\mathbf {u}}_i$ that appears non-physical on the first glance may be combined with one or more of the other ${\mathbf {u}}_i$ to obtain a physical solution (more detailed explanation will follow). Nevertheless, all those ${\mathbf {u}}_i$ have to display a corresponding singular value equal to zero. A way to obtain the physical solutions from the solution vectors ${\mathbf {u}}_i$ is given in the “Methods” section.

Now, a helpful twist to the argument is made. In the previous explanation, $N_0$ singular values were assumed to be zero. This assumption does not necessarily prove true as the determinant of ${\mathbf {M}}$ is not even close to zero in most cases and may easily exceed $\text {det}({\mathbf {M}})>10^4$ (assuming several 10,000 grid nodes). This behaviour is no coincidence: The density function at the edge of the domain has to be set to zero in a similar fashion as in the RF phase modulation case. The domain edge being set to zero will cause the full destruction of the beam in the temporal asymptotic limit due to the diffusion in the system. It therefore follows that only the trivial zero solution to Eq. (14) can exist as an equilibrium state. Nevertheless, we are interested in those solutions ${\mathbf {u}}_i$ that correspond to the smallest singular values and are in this sense the solutions that are the closest to an equilibrium state. These momentary equilibrium states may have a sufficient lifetime to be observed in the machine. It turns out handy that for a large enough domain the condition number $\kappa$ becomes large even though the determinant exceeds, e.g., $10^4$. The condition number $\kappa =\sigma _\text {max}/\sigma _\text {min}$ is the ratio of the largest ($\sigma _\text {max}$) and smallest singular value $(\sigma _\text {min})$, usually indicating an ill-conditioned inverse of ${\mathbf {M}}$ if $\kappa$ is large. In our case, a sparse matrix ${\mathbf {M}}$ with a large $\kappa$ means that at least one solution exists with a corresponding small singular value even when the determinant is large, thus providing us with a suitable equilibrium state to the VFP equation.

Until now, it has been tacitly assumed without explicit proof that a vector ${\mathbf {u}}_i$ obtained from the SVD (see Eq. 15) with a corresponding small relative singular value is more or less an equilibrium state that does not change over time. The correctness of this assumption can be derived by re-introducing the time-dependence in Eq. (11) into the discretized VFP equation in Eq. (14) and substituting ${\mathbf {x}}={\mathbf {u}}_i$

17 $$\begin{aligned} {{\mathbf {M}}}{{\mathbf {u}}}_i=\frac{\partial {\mathbf {u}}_i}{\partial t}. \end{aligned}$$

A simple integration in time can be achieved via the explicit Euler method that would lead to the modification of the density vector ${\mathbf {u}}_i$ by adding the difference vector $\Delta {\mathbf {u}}_i = \Delta t {{\mathbf {M}}}{{\mathbf {u}}}_i$ in the integration step with $\Delta t$ being the step size in terms of time. From the well-known properties of the SVD one directly obtains the relation

18 $$\begin{aligned} \frac{ ||\Delta {\mathbf {u}}_i||}{ ||{\mathbf {u}}_i||} = \Delta t \sigma _i \end{aligned}$$

for the 2-norms of the increment vector and the state vector. Equation (18) indicates that the 2-norm of the difference vector is negligible even in the presence of a large integration step in time in comparison to the original state vector once the singular value becomes sufficiently small.

In the context of the solutions to Eq. (14), the question remains what a small singular value and large enough grid size is. To investigate this, let us assume one finds a solution vector ${\mathbf {x}}$ for Eq. (14). Multiplying ${\mathbf {x}}$ with a scalar $\alpha$ leaves $\alpha {\mathbf {x}}$ in the kernel. This is equivalent to scaling the bunch intensity that does not influence the bunch length or shape since collective effects are neglected and radiation damping as well as quantum excitation are single-particle processes. Hence,

19 $$\begin{aligned} {\mathbf {M}}(\alpha {\mathbf {x}})={\mathbf {0}} \end{aligned}$$

is also fulfilled besides Eq. (14). Substituting the SVD from Eq. (15) into Eq. (19) and rearrangement leads to

20 $$\begin{aligned} {\mathbf {V}} (\alpha {\Sigma }){\mathbf {U}}^\mathrm {T} {\mathbf {x}}={\mathbf {0}}. \end{aligned}$$

This demonstrates that the scalar $\alpha$ can be moved onto the singular values in ${\Sigma }$. Consequently, only the ratio between the singular values with respect to the largest one matters since the scaling can make them arbitrarily small without changing the dynamics of the system (the condition number $\kappa$ of the matrix remains unchanged).

### Overlapping versus disjoint islands

**Figure 4:**
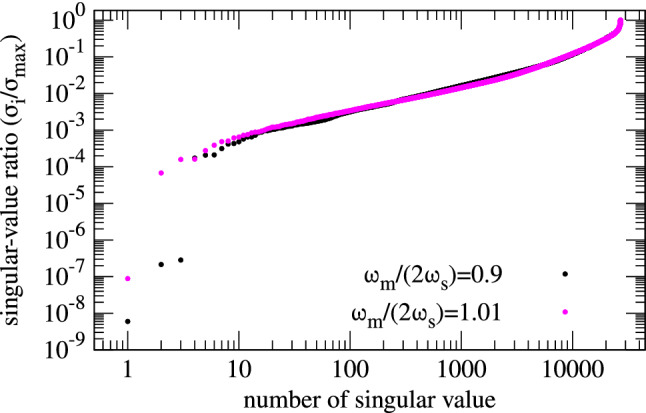
Sorted singular values normalized to the largest one. These singular values arise from an RF phase modulation scenario with $n=2$, $A_\text {m}\tan \phi _\text {s}=0.1$ and $\omega _\text {m}/(2 \omega _\text {s})=0.9$ (black) and $\omega _\text {m}/(2 \omega _\text {s})=1.01$ (magenta) on a $308 \times 88$ grid. In the $\omega _\text {m}/(2 \omega _\text {s})=0.9$ case, the first three singular values show a much lower ratio in the range of $\sigma _i/\sigma _\text {max}<10^{-6}$ and are therefore considered the physical equilibrium solutions of the VFP equation, since there is a step of more than two orders of magnitude to the next singular value that has $\sigma _i/\sigma _\text {max}\approx 10^{-4}$. In the $\omega _\text {m}/(2 \omega _\text {s})=1.01$ case, there is only one equilibrium state with $\sigma _i/\sigma _\text {max} \ll 10^{-4}$.

In this section, the difference between independent (disjoint) islands and those that interact via synchrotron radiation damping and quantum excitation is elaborated using the example of RF phase modulation at the second integer resonance. The third integer resonance is going to be analyzed in the context of transverse resonance island buckets in a separate section. The following example is carried out with the parameters $A_\text {m}\tan \phi _s =0.1$ and $\omega _\text {m}/(2\omega _\text {s})=0.9$ (three-island regime) on a $N_J \times N_\Psi =308 \times 88$ grid. The damping constant is here $\gamma _\text {d} ={260}{\hbox {s}^{-1}}$ and the bunch length measured in terms of the RF phase angle is $\sigma _z = {0.16}\hbox {rad}$. The ratio of the singular values to the maximum singular value of the equilibrium solutions is in the range of $10^{-9}$ to $10^{-6}$ as is displayed in Fig. 4.

**Figure 5:**
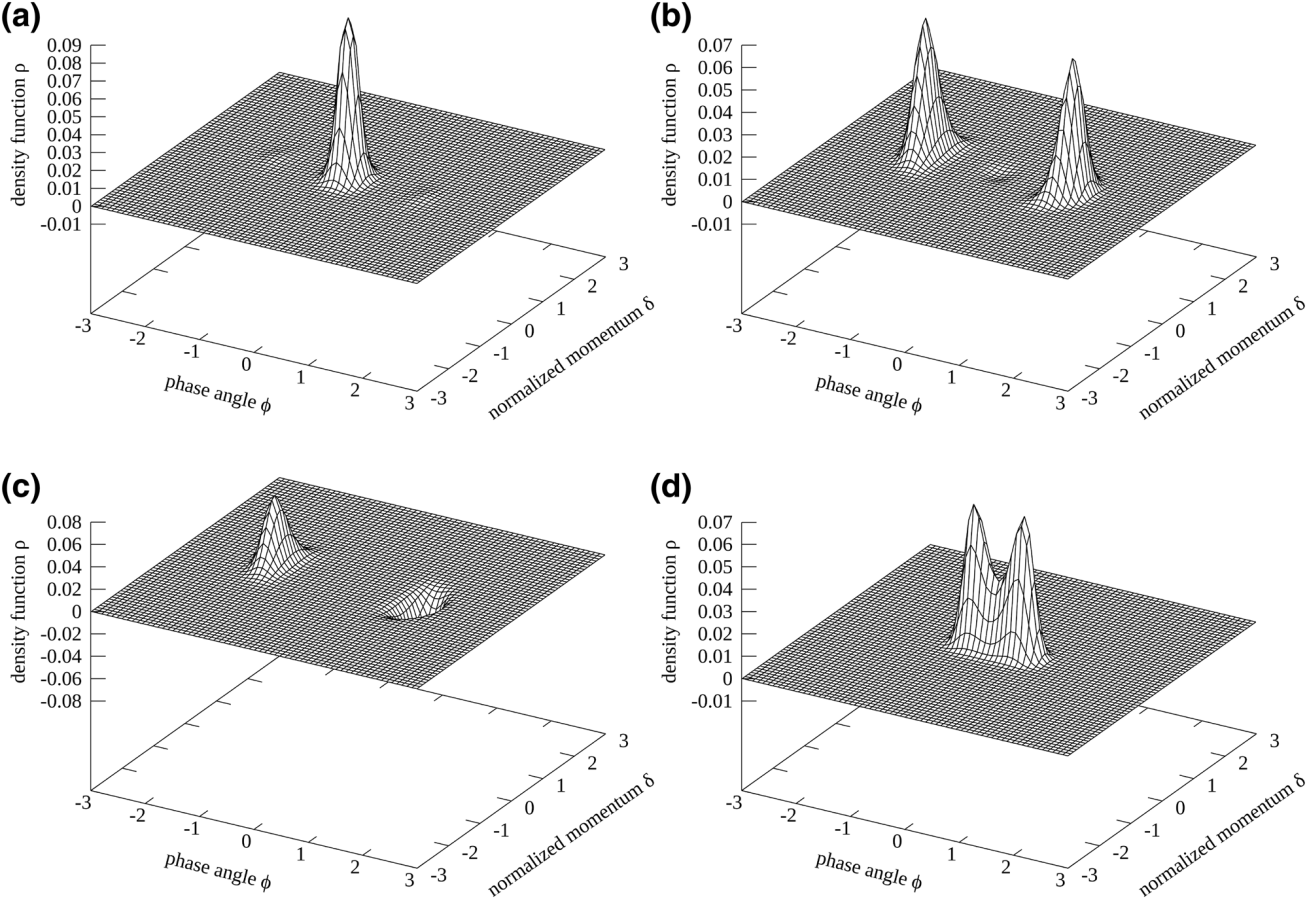
The base vectors ${\mathbf {u}}_1$ **(a)**, ${\mathbf {u}}_2$ **(b)**, ${\mathbf {u}}_3$ **(c)** for the RF phase modulation scenario with $n=2$, $A_\text {m}\tan \phi _s =0.1$ and $\omega _\text {m}/(2\omega _\text {s})=0.9$. Although ${\mathbf {u}}_2$ and ${\mathbf {u}}_3$ display partly negative density values, linear combinations of the three base vectors yield three disjoint islands with exclusively positive density values. **(d)** Equilibrium state ${\mathbf {u}}_1$ for the RF phase modulation scenario with $n=2$, $A_\text {m}\tan \phi _s =0.1$ and $\omega _\text {m}/(2\omega _\text {s})=1.01$. This is the only equilibrium state since the two islands are connected via a ridge and therefore depend on each others’ densities.

In this particular case (black dots), there are three equilibrium solutions (the fourth singular value is separated by a step of more than two orders of magnitude). The respective solution vectors ${\mathbf {u}}_i$ are depicted in Fig. 5a–c.

Although ${\mathbf {u}}_2$ and ${\mathbf {u}}_3$ display partly negative $\rho$ values, a sum ${\mathbf {x}}=\sum _{i=1}^3 b_i {\mathbf {u}}_i$ can be found that yields solely positive $\rho$ values and therefore there are three valid equilibrium states. Negative $\rho$ values are produced due to the choice of basis vectors for the solution space. Rotating the basis of the kernel would lead to exclusively positive $\rho$ values; however, this is not of interest in the following. There are three solutions since the three islands are disjoint, i.e., not connected via particle transfer due to radiation damping or diffusion, and the vectors ${\mathbf {u}}_i$ can be summed in a way that only a single island exists (either one of the lateral islands or the centre island). To give an example of overlapping islands, the only existing equilibrium solution for $A_\text {m}\tan \phi _s =0.1$ and $\omega _\text {m}/(2\omega _\text {s})=1.01$ (two-island regime) is given in Fig. 5d, i.e., the two islands will always have the same shape and intensity and the islands cannot exist independently of each other since a ridge connects them. The ratio of the singular values for this particular scenario is also given in Fig. 4 (magenta markers).

### Dependence of the equilibrium states at $\mathbf {n=2}$ on the modulation frequency

**Figure 6:**
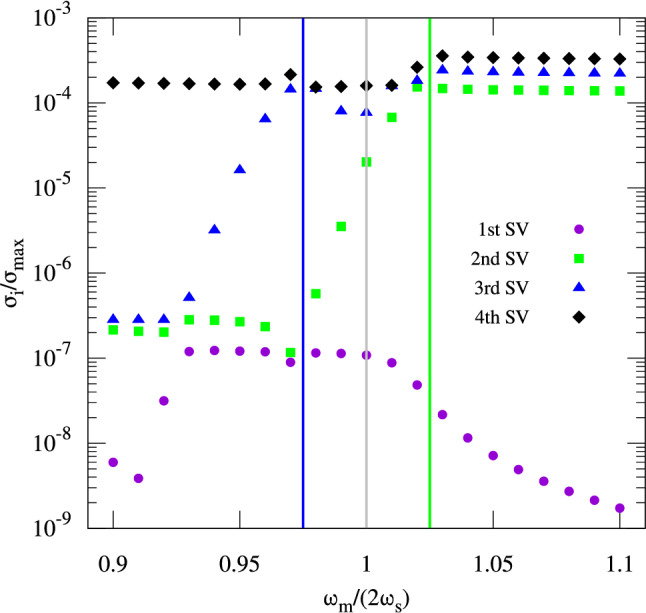
Ratio between the four smallest singular values (SVs) $\sigma _i$ and the largest one $\sigma _\text {max}$ under the variation of the modulation frequency of the phase modulation scenario with $n=2$ and $A_\text {m}\tan \phi _\text {s}=0.1$ on a $308 \times 88$ grid. Below the upper bifurcation frequency (green line), the second smallest singular value (green squares) starts to decrease since the lateral islands drifts away from the centre and the two islands become disjoint at $\omega _\text {m}/(2\omega _\text {s})\approx 0.97$. Below the lower bifurcation frequency (blue line), the third singular value (blue triangles) starts to decrease since the islands in the three-island regime become more and more disjoint towards $\omega _\text {m}/(2\omega _\text {s})\approx 0.93$, where all three states show roughly the same magnitude in terms of singular value compared to the fourth singular value (black diamonds), which constitutes the first non-physical mode remaining at large amplitude independently of the modulation frequency.

In the following, the base vectors ${\mathbf {u}}$ of the kernel of ${\mathbf {M}}$ are analysed for $n=2$. For illustrative purposes, a scan of the modulation frequency is performed with constant modulation amplitude ($A_\text {m}\tan \phi _s = 0.1$). This scan is also shown in the Supplementary Video S1. This analysis for different modulation frequencies $\omega _\text {m}/(2 \omega _\text {s})$ studies the behaviour of the singular values corresponding to the equilibrium solutions at the bifurcation frequencies and identifies the frequencies at which islands become disjoint from each other. The latter points are identified by the locations where the singular values of the equilibrium states ${\mathbf {u}}$ have reached roughly the same amplitude and are clearly distinguishable from the non-physical solution states that ideally have singular values several orders of magnitude larger than the equilibrium states. The evolution of the singular values during the scan of the modulation frequency is shown in Fig. 6. Below each of the two bifurcation frequencies $\omega _\text {m}/(2\omega _\text {s})=1.025$ (green line) and $\omega _\text {m}/(2\omega _\text {s})=0.975$ (blue line), one additional singular value starts to decrease towards smaller modulation frequencies. The second singular value reaches the level of magnitude of the first singular value at $\omega _\text {m}/(2\omega _\text {s})=0.97$. This indicates that the two lateral islands have become disjoint and the centre island has vanished. Consequently, long-term stability of the lateral islands can be expected in the limit of the approximations made within the presented framework. The third singular value reaches a similar level as the other two at $\omega _\text {m}/(2\omega _\text {s})=0.93$, while the first singular value is again decreasing for even lower modulation frequencies. Here, three islands exist with both lateral islands having become disjoint from the centre one. Hence, the three states are expected to have comparable lifetimes in terms of diffusion (does not necessarily refer to the beam lifetime). The main reason for the significantly asymmetric behaviour observed in the plot around the resonance frequency at $\omega _\text {m}/(2\omega _\text {s})=1$ (grey line) is the distance of the stable fixed points from the bunch centre ($J=0$). Following Eqs. (7)–(8), the action of the stable fixed points vanishes at the upper bifurcation frequency and increases towards lower modulation frequencies.

It is important to note that one does not see a rapid jump of the smallest singular values at the crossing of a bifurcation frequency, since the stable fixed points move apart slowly in terms of action. At the bifurcation frequencies, additional stable fixed points appear or disappear in immediate vicinity of each other causing an overlap of beamlets. As an example, one may look at a bifurcation at which a stable fixed points splits into two stable fixed points ($\omega _\text {m}/(2\omega _\text {s})\approx 1.025$ in Fig. 6). Right after the bifurcation has taken place, the two stable fixed points are so close to each other that the resulting density function is indistinguishable from the state just before the bifurcation has taken place (a single stable fixed point), and the two islands are only slowly moving apart to finally become disjoint for $\omega _\text {m}/(2\omega _\text {s})\approx 0.97$. This behavior is not a result of the applied approximation or concept; instead, it is a physical feature that is observed in a real storage ring.

## Transverse resonance islands

The previous sections dealt with the longitudinal phase-space distribution under the influence of an RF phase modulation. Surprisingly, the Hamiltonian of the transverse phase space when the storage ring is operated on an optical resonance at, e.g., the horizontal tune being $Q_x=1/3$, is nearly identical. In the following, the symbol $Q_x$ will only refer to the fractional part of the tune. These resonances in the transverse planes are driven in first order by the lattice sextupoles, leading to a Hamiltonian that is analogous to that for RF phase modulation in the longitudinal plane with $n=3$. Of course, the dimensions of the longitudinal plane and the transverse planes as well as the sources of excitation are highly different. Nevertheless, a one-to-one correspondence between the parameters in the different planes can be made as is demonstrated in the following.

Operating a synchrotron or storage ring on a transverse resonance to achieve a transversely split beam (transverse resonance islands) may have many reasons. At synchrotron light sources, radiation with different helicity may be achieved due to the different transverse angles $x'$ of the beamlets in elliptical undulators^3^. At hadron accelerators like the PS at CERN (see, e.g., Ref.^5^), a multi-turn extraction with transversely split beams is performed to artificially enlarge the PS circumference in terms of the extracted bunch train (resonant orbit closes after multiple turns).

### The Hamiltonian in the horizontal plane with an arbitrary sextupole distribution

In the following, the vertical plane is neglected from the analysis of the transverse phase space. The vertical tune is ideally placed far away from an optical resonance and sufficiently integrable. The Hamiltonian in the transverse plane in action angle coordinates $(\psi _x,I_x)$ in the storage ring then reads $H(I_x) = \mu _x^C I_x$ with the total horizontal phase advance $\mu _x^C = \int _C \text {d}s/\beta _x(s)$, the horizontal beta function $\beta _x$, and the horizontal action $I_x$. The Hamiltonian

21 $$\begin{aligned}H(I_x,\psi _x,s_0) = \mu _x^C I_x + \mu _x^C \sum _i^{N_\text {s}} \frac{S_i (2\beta _x(s_i)I_x)^{3/2}}{8} \nonumber \\&\quad \left( \frac{\cos \left( 3( \psi _x + \mu _x(s_i)-\mu _x(s_0) -\mu _x^C /2) \right) }{\sin \left( 3 \mu _x^C /2 \right) } \right. \left. + \frac{\cos \left( \psi _x + \mu _x(s_i)-\mu _x(s_0) -\mu _x^C /2 \right) }{\sin \left( \mu _x^C /2 \right) } \right) \, \end{aligned}$$

can be derived using Lie algebra up to first order in the sextupole strength and in the approximation of thin sextupoles^14^. Here, $N_\text {s}$ is the number of sextupoles, $S_i$ is the integrated sextupole strength of the *i*th sextupole at location $s_i$ defined via the integrated kick of a sextupole $\Delta x'= S_i x^2$ in thin-lens approximation (different definition for $S_i$ in Ref.^14^), $\mu _x(s)=\int _0^s \mathrm {d}s/\beta _x(s)$ is the phase advance at location *s*, and $s_0$ is the point of observation along the circumference. Equation (21) indicates that integer resonances as well as 1/3 resonances are driven in first order by sextupoles since at least one of two sine functions in the denominators approaches zero in these cases.

The Hamiltonian in Eq. (21), however, lacks amplitude detuning that is required for deriving (stable) fixed points in phase space, since sextupoles generate amplitude detuning only in second order of the sextupole strength. That second-order effect therefore vanishes in the first-order approximation previously made. For this reason, the linear detuning parameter in the horizontal plane $\alpha _{xx} = \text {d} Q_x/\text {d} I_x$ is artificially supplemented to the Hamiltonian. The parameter $\alpha _{xx}$ comprises first-order octupole and second-order sextupole contributions. The respective expressions can be found in the standard literature, e.g., Reference^15^. In the following, only the linear detuning contribution will be considered; however, the presented formalism and the approach of solving the VFP equation are not restricted to the linear detuning term and high-order terms can be supplemented to achieve better accuracy if desired.

The Hamiltonian at the third-order resonance, i.e., $3\mu _x^C =n 2\pi +3\varepsilon$, with $n \in {\mathbb {Z}}^+$ and a small offset $\varepsilon$, signifying the resonance-proximity parameter, reduces to

22 $$\begin{aligned}H (I_x,\Psi _x,s_0)= \mu _x^C I_x + \pi \alpha _{xx} I_{x}^2 + \mu _x^C \sum _i^{N_\text {s}} \frac{S_i (2\beta _x(s_i)I_x)^{3/2}}{8\sin (3\mu _x^C /2)} \cos \nonumber \\&\quad \left[ 3\left( \psi _x + \mu _x(s_i)- \mu _x(s_0) -\frac{\mu _x^C}{ 2} \right) \right] . \end{aligned}$$

In order to find the fixed points, a transformation into a rotating reference frame is once again required as was performed to obtain a time-independent Hamiltonian for the RF phase-modulation case. To do so, the phase advance at the observation point $\mu _x(s_0)$ is going to rotate with the tune $Q_x=1/3$ of the nearby resonance, i.e., $\mu _x(s_0)=2\pi n \tau /3$ with the time-like variable $\tau$. This requires a coordinate transformation of $(\psi _x,I_x)$: The generating function $F_2=(\psi _x-2\pi n \tau /3-\mu _x^C /2)I_x$ results in the substitutions ${\phi }_x=\psi _x-2\pi n \tau /3-\mu _x^C /2$ and $J_x=I_x$. With the generating function and these substitutions, Eq. (22) transforms into the location-independent Hamiltonian

23 $$\begin{aligned} K(J_x,\phi _x)= \varepsilon J_x + \pi \alpha _{xx} J_{x}^2 + \mu _x^C \sum _i^{N_\text {s}} \frac{S_{i}(2\beta _{x}(s_i)J_x)^{3/2}}{8 \sin (3\mu _x^C /2)} \cos \left( 3 \phi _x +3 \mu _x(s_i) \right) . \end{aligned}$$

This Hamiltonian still contains a sum over all sextupoles; however, since the argument of the cosine functions is the same among all terms with the exception of the phases $3 \mu _x(s_i)$, the sum can be combined into a single cosine function

24 $$\begin{aligned} K(J_x,\phi _x)= \varepsilon J_x + \pi \alpha _{xx} J_{x}^2 + A_\mathrm {t} J_x^{3/2} \cos \left( 3 \phi _x + 3 \phi _{x0} \right) \, \end{aligned}$$

with the amplitude $A_\mathrm {t}$ and the phase $\phi _{x0}$ which is set to zero in the following by redefinition of $\phi _x$. The explicit transformation to obtain Eq. (24) from Eq. (23) follows

25 $$\begin{aligned} A_\mathrm {t}\cos (3\phi _x + 3 \phi _{x0}) = \left[ \left( \sum _i^{N_s} a_i \cos 3\varphi _i \right) ^2+\left( \sum _i^{N_s} a_i \sin 3\varphi _i \right) ^2 \right] ^{\frac{1}{2}} \cos \left[ 3\phi _x + \arctan \left( \frac{\sum _i^{N_s} a_i \sin 3\varphi _i) }{\sum _i^{N_s} a_i \cos 3\varphi _i) } \right) \right] \end{aligned}$$

with $$\varphi _i=\mu _x(s_i)$$ and $$a_i=\mu _x^C S_i (2 \beta _{x}(s_i))^{3/2}/({8 \sin (3\mu _x^C /2)})$$.

The one-to-one correspondence of the Hamiltonian in Eq. (24) with respect to the Hamiltonian of the RF phase modulation in Eq. (3) is clearly visible: In both cases, there is a type of resonance-proximity parameter ($\varepsilon$ and $\omega _\text {s}/(m \omega _\text {m})$, respectively), detuning with increasing particle action ($\alpha _{xx}$ and $\omega _s/16$, respectively) and some sort of modulation amplitude ($A_\mathrm {t}$ and $A_\text {m}$, respectively). The newly introduced amplitude $A_\mathrm {t}$ is a function of the sextupole resonance driving term (see, e.g., Refs.^16,17^)

26 $$\begin{aligned} h_{3000} \propto \sum _i^{N_\mathrm {s}} S_i \beta _x^{3/2}(s_i) \exp (i3\mu _x(s_i))\, \end{aligned}$$

that dwarfs all other resonances at $Q_x\approx 1/3$. The index of $h_{3000}$ describes the dependence of the driving term on the two transverse actions $J_{x,y}$ and the phases $\mu _{x,y}$. For a more detailed explanation, the reader is referred to, e.g.,^16,17^. The Eqs. (25) and (26) demonstrate the absence of lateral islands in a fully periodic accelerator lattice at $Q_x\approx 1/3$ since the sum and therefore the amplitude $A_\mathrm {t}$ cancels out if the sextupoles are distributed periodically with the cell period which is usually the case to suppress resonance driving terms. Breaking the symmetry to achieve a non-vanishing magnitude of the $h_\mathrm {t}$ driving term is therefore required. The stability of the fixed points as well as the bifurcation diagram correspond to the expressions given in Eqs. (9)–(10) for the third integer resonance in the case of RF phase modulation with the respective substitutions.

### Dependence of the equilibrium states at $\mathbf {Q_x=1/3}$ on the modulation amplitude

The VFP in the transverse plane corresponds to the VFP in the longitudinal plane with the exception of different parameters in the Fokker-Planck collision term: The horizontal damping rate is usually half as large as the longitudinal one and consequently the diffusion coefficient $D_x$ is different due to the different damping rate and equilibrium emittance. Nevertheless, the VFP remains otherwise unchanged and the discretization in Eq. (13) is applicable. In the following, a fractional betatron tune of $Q_x=0.305$ (the resulting resonance-proximity parameter is $\varepsilon =2 \pi (Q_x-1/3)=-0.178$), a bunch size of $\sigma _x=1.0\times 10^{-4}\hbox {m}$ at $\beta _x={1}\hbox {m}$, $\alpha _{xx}=6.7\times 10^{4}{\hbox {m}^{-1}}$ and a ratio of the damping factor $\gamma _\text {d}$ to the revolution frequency $f_0$ of $\gamma _\text {d}/f_0={5.0\times 10^{-5}}$ is assumed. Since the time-like variable $\tau$ is a measure of the turn number, the normalization of the damping parameter $\gamma _\text {d}={130}{\hbox {s}^{-1}}$ to the revolution frequency $f_0={2.6}\hbox {MHz}$ is required. These parameters are typical for the 1.5-GeV synchrotron light source DELTA at the TU Dortmund University that is taken here as an example. The VFP is solved on a $400\times 80$ grid (32,000 grid nodes) with cyclic boundary conditions on 1/3 of the domain in terms of the angle $\phi _x \in [ 0; 2\pi /3[$, exploiting the three-fold rotational symmetry of the system.

### Analysis of disjoint transverse islands

Exploiting the rotation symmetry, i.e., restricting the angle to $\phi _x \in [ 0; 2\pi /3[$, reduces the number of potential disjoint modes to two since only the centre islands and one lateral island are covered while two lateral islands are outside the $\phi _x$ range. Solving the VFP for $2 \pi$, would enable the possibility of four disjoint modes (four potential islands in the domain).

**Figure 7:**
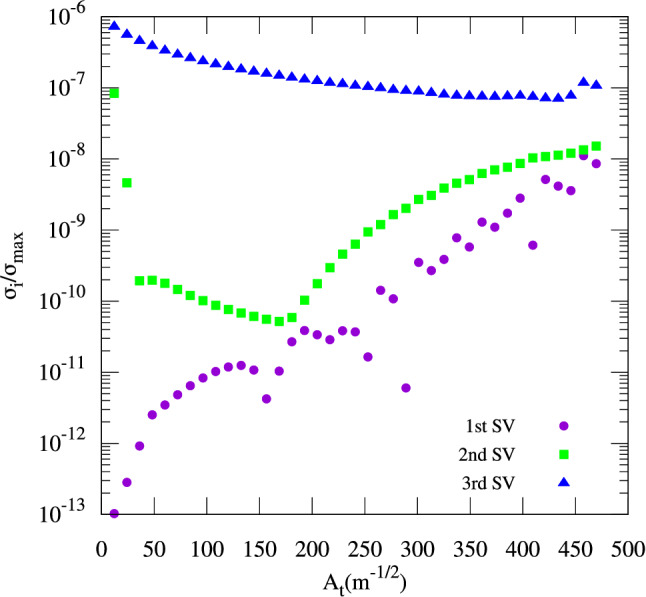
Ratio between the three smallest singular values $\sigma _i$ and the largest singular value $\sigma _\text {max}$ versus the modulation amplitude $A_\mathrm {t}$ ($\alpha _{xx}=\mathrm {const.}$) obtained by solving the VFP equation with $Q=0.31$ on a $400 \times 80$ grid (three-fold rotation symmetry exploited). At $A_\mathrm {t}={181}{\hbox {m}^{-1/2}}$, the two smallest singular values (purple circles and green squares) have roughly the same magnitude while the third smallest one remains comparably large (blue triangles) as a reference.

A scan of the modulation amplitude $A_\mathrm {t}$ is performed in the following while the linear detuning $\alpha _{xx}$ is held constant during this procedure. The scan is shown in Fig. 7. An increase of the modulation amplitude drives the lateral islands away from the bunch center. At small amplitudes $A_\mathrm {t}< {100}{\hbox {m}^{-1/2}}$, the radiation damping has to be overcome by the excitation and the SFPs have to move out of the bunch core before stable lateral islands can be observed. Hence, only a single equilibrium solution is observed early on ($\sigma _1 \ll \sigma _2$).

**Figure 8:**
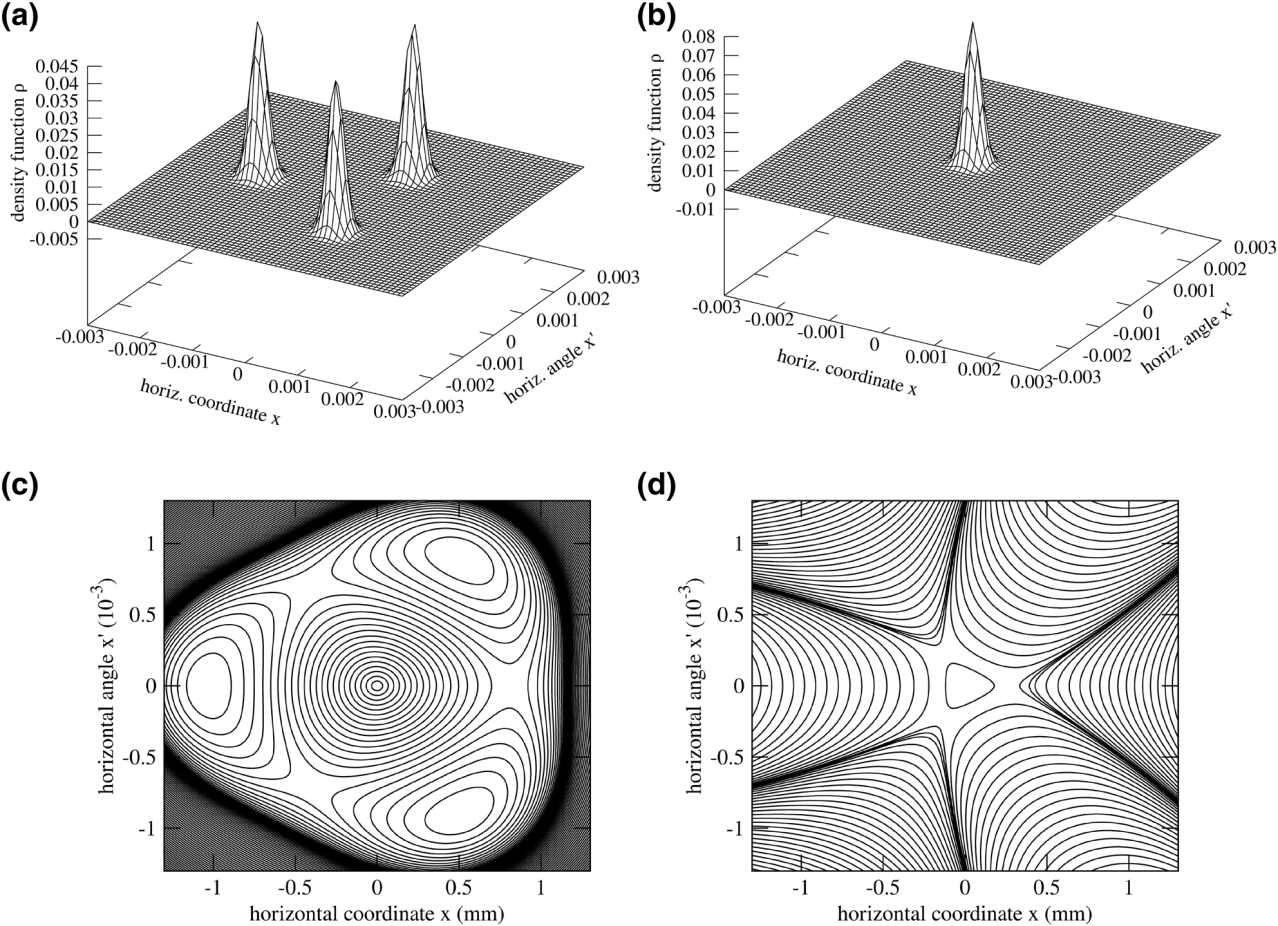
**(a)** Example of a physical solution vector obtained by linear combination of the two base vectors ${\mathbf {x}}_{1}=0.77{\mathbf {u}}_1+0.63{\mathbf {u}}_2$ for the $Q=0.305$ scenario with $A_\mathrm {t}= {181}{\hbox {m}^{-1/2}}$ with $\beta _x={1}\hbox {m}$. The domain is artificially filled up since only the angle $\phi _x\in [0;\,2\pi /3[$ has been calculated. **(b)** The second physical solution vector obtained via the linear combination ${\mathbf {x}}_{2}=-0.63{\mathbf {u}}_1+0.77{\mathbf {u}}_2$. Note that ${\mathbf {x}}_1$ and ${\mathbf {x}}_2$ are orthogonal. **(c)** Contour lines of a constant Hamiltonian *K* (no constant increment of *K* between contour lines) for $A_\mathrm {t}= {44}{\hbox {m}^{-1/2}}$. **(d)** Contour lines of a constant Hamiltonian *K* for $A_\mathrm {t}= {583}{\hbox {m}^{-1/2}}$ with $\beta _x={1}\hbox {m}$. The available dynamic aperture for the bunch core is much smaller in this picture compared to the one on the left. Here, multiple standard deviations of the beam size $\sigma _x={1.0\times 10^{-4}}\hbox {m}$ no longer fit into the stable region.

Once the amplitude increases, the action of the stable fixed points also increases. At $A_\mathrm {t}= {181}{\hbox {m}^{-1/2}}$, the lateral islands become disjoint from the centre islands, i.e., the modes corresponding to the centre island and to the lateral islands display roughly equal singular values in terms of magnitude. The two possible orthogonal solution vectors that have exclusively positive density values are shown in Fig. 8a,b. When both modes have become disjoint, it is expected that a minimum amount of particles will travel between the bunch core and the lateral islands and they should roughly share the same lifetime in terms of diffusion. Choosing this particular modulation amplitude may be the optimal mode of operation since maximum stability of the transverse resonance islands can be inferred.

Above $A_\mathrm {t} = {181}\,{\hbox {m}^{-1/2}}$, the singular values corresponding to the lateral islands increase once again since the lateral islands drift towards the domain edge. Towards large amplitudes ($A_\mathrm {t}> {300}\,{\hbox {m}^{-1/2}}$), also the singular value corresponding to the bunch core increases since the dynamic aperture for the bunch core shrinks. To demonstrate the shrinkage of the separatrix, Fig. 8c,d shows contour lines of the Hamiltonian *K* for $A_\mathrm {t}={44}\,{\hbox {m}^{-1/2}}$ and for $A_\mathrm {t}={583}\,{\hbox {m}^{-1/2}}$. The radius of the stable region around the coordinate origin has decreased by roughly a factor of three. With $A_\mathrm {t}>{300}\,{\hbox {m}^{-1/2}}$, multiple standard deviations of the beam size $\sigma _x={1.0\times 10^{-4}}\,\mathrm{m}$ no longer easily fit into the stable region. Consequently, an increased singular value for the mode corresponding to the bunch core is observed since particles diffuse out of the core.

## Conclusion

Throughout this paper, a numerical solution using a finite-differences approach to the VFP equation has been described. A subtle indicator, i.e., the magnitude of the singular values of the matrix, has been introduced to infer the long-term stability of the steady-state solutions of the VFP equation under the influence of quantum excitation and radiation damping in the longitudinal phase space (RF phase modulation) and the transverse phase space (transverse resonance islands). The approach avoids tracking studies that are not only time-consuming, but also cannot reliably identify all different equilibrium states since the number of simulated turns and the initial distribution have major effects on the final outcome. The concept of disjoint and overlapping lateral islands in both the longitudinal and transverse phase space has been introduced and successfully demonstrated with the examples of the second integer resonance for RF phase modulation and the sextupole resonance at $Q_x\approx 1/3$ in the transverse phase space. This approach also allows to determine a setup with the smallest amount of particle leakage between the different islands.

Although simplifying approximations have been made, this novel approach of inspecting the singular values to infer long-term stability may prove handy even when the system complexity increases by, e.g., the inclusion of high-order detuning terms. The only requirement is the successful derivation of the time-independent Hamiltonian to avoid solving the VFP in the temporal domain. Overall, with the outlined formalism the general problem of long-time stability for novel bunch filling patterns in modern electron storage rings may become more accessible for a quick theoretical assessment. Thus, the search for innovative operational schemes at fourth-generation synchrotron facilities like MAX IV^1^ or ESRF-EBS^2^ could be relieved and completely new design lattices be explored, opening the door to future even more powerful and specialized X-ray machines.

## Methods

### Obtaining the physical solutions

As previously mentioned, the obtained solution vectors ${\mathbf {u}}_i$ are not necessarily identical with the actual physical solutions, since they may display density values smaller and larger than zero and depend on the algebraic software that is used (SVD is non-unique). We only consider cases in which there is at least one singular value sufficiently small with respect to the larger singular values, since otherwise there will be no equilibrium states to hold on to any stored beam. Hence, $N_0 \geqslant 1$ singular values that are at least a few orders of magnitude smaller than the next larger one are assumed from now on. Such a system has to feature $N_0$ beamlets because of symmetry reasons (Since the Hamiltonian is symmetric in $\Psi$, either all or none of the islands overlap. If they all overlap, there is only a single distinct singular value that is sufficiently small ($N_0=1$). This single solution vector has to be physical in any case or the beam cannot be stored otherwise). Each of the solution vectors ${\mathbf {u}}_i$ is a superposition of the physical solutions ${\mathbf {e}}_j$ we want to solve for. This can be written in matrix form

27 $$\begin{aligned} {\mathbf {U}}={{\mathbf {C}}}{{\mathbf {E}}} \end{aligned}$$

with ${\mathbf {U}}$ containing the solution vectors ${\mathbf {u}}_i$ from the SVD row-wise, ${\mathbf {C}}$ being an orthonormal $N_0\times N_0$ coefficient matrix and ${\mathbf {E}}$ containing the physical solutions we want to solve for row-wise. Solving directly for ${\mathbf {E}}$ is not possible since ${\mathbf {C}}$ is unknown; however, there is an intuitive way to obtain the transformation: Each of the solution vectors ${\mathbf {u}}_i$ will display positive and/or negative peaks at the center of each of the beamlets (see, e.g., Fig. 5). For each ${\mathbf {u}}_i$, one can construct a new vector $\hat{{\mathbf {u}}}_i=k_i(\rho _{i,1}, \dots , \rho _{i,N_0})$ with $\rho _{i,j}$ being the density value of the corresponding ${\mathbf {u}}_i$ vector read from the grid at the location of the peak (may it have positive or negative sign) of the *j*th island, and $k_i$ being a scalar that ensures a unit vector length. The idea behind the construction of these vectors is that the $\rho _{i,j}$ is proportional to the contribution of ${\mathbf {e}}_j$ to ${\mathbf {u}}_i$, i.e., $\rho _{i,j}\propto {\mathbf {C}}_{i,j}$. With the matrix ${\hat{\mathbf {C}}}=(\hat{\mathrm {u}}_1, \dots , \hat{\mathrm {u}}_{N_0})^\mathrm {T}$, one can calculate its inverse ${\hat{\mathbf {C}}}^{-1}$. The matrix ${\hat{\mathbf {C}}}^{-1}$ is equal to ${\mathbf {C}}^{-1}$ after restoring unit length of the row vectors of ${\hat{\mathbf {C}}}^{-1}$. As a last step, the simple formula ${\mathbf {E}}={\mathbf {C}}^{-1}{\mathbf {U}}$ yields the physical solutions.

## Supplementary Information

Supplementary Legends.

Supplementary Video S1.

## Data Availability

The datasets that were produced, analyzed and presented during the current study are available from the corresponding author on reasonable request.
