# Supplementary figures and images for: Steady-state solutions of split beams in electron storage rings
Source: Sci Rep. 2022 Nov 1;12:18383. doi: 10.1038/s41598-022-22857-y (PMC9626505; doi:10.1038/s41598-022-22857-y)

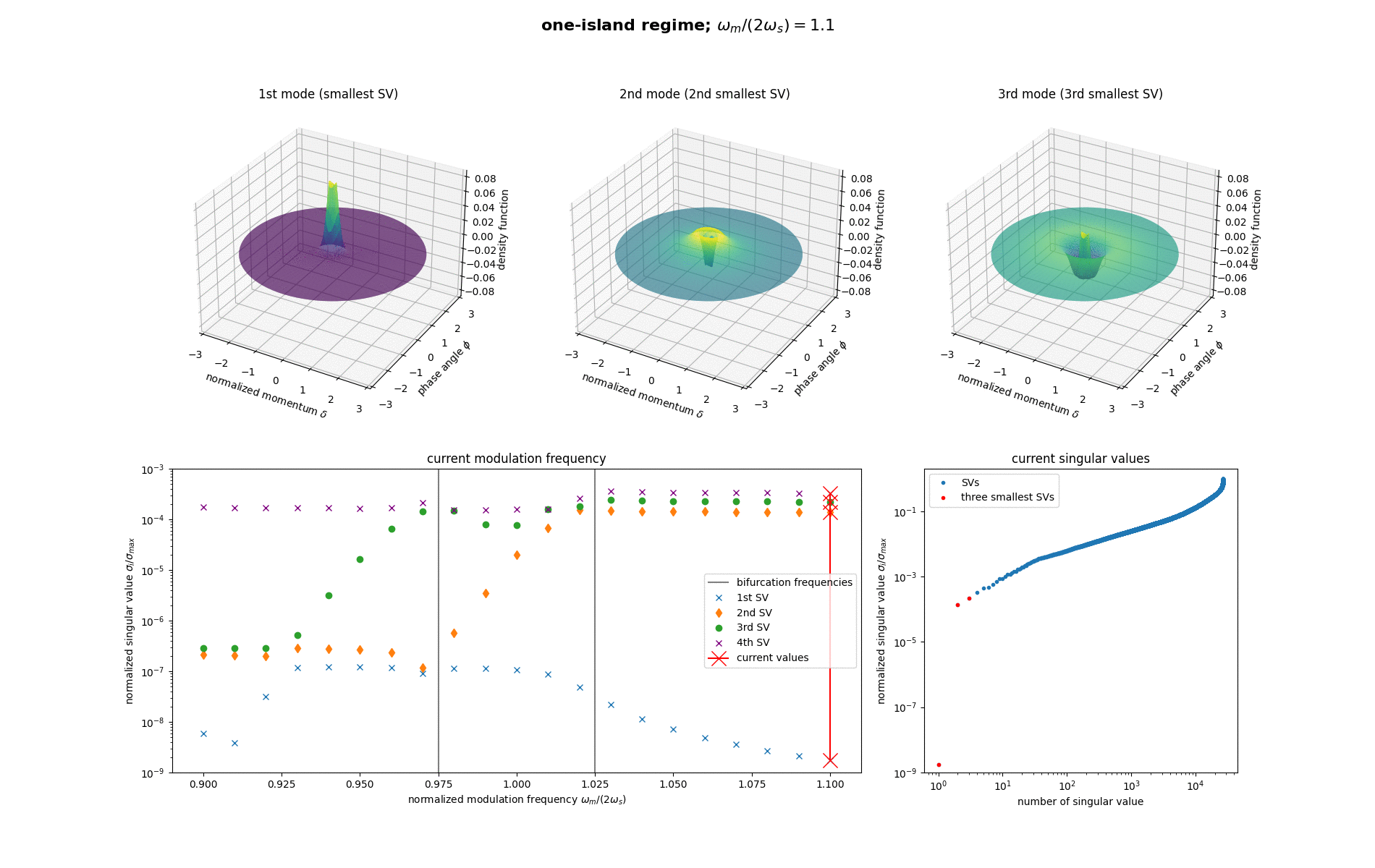

Supplement: Supplementary file 2 — Supplementary Video S1. [file 41598_2022_22857_MOESM2_ESM.gif]
